# Novel Unbalanced Translocation t(3;13)(q29;q34) in an Infant With Hydrocephalus

**DOI:** 10.1002/bdr2.2517

**Published:** 2025-08-08

**Authors:** Teresa E. Fowler, Anthony R. Gregg, Ryan F. Bloomquist

**Affiliations:** ^1^ Department of Ophthalmology Wellstar‐MCG Health Augusta Georgia USA; ^2^ Department of Obstetrics and Gynecology Prisma Health Richland Hospital Columbia South Carolina USA; ^3^ University of South Carolina School of Medicine Columbia South Carolina USA

**Keywords:** 13q34, 3q29, hydrocephalus, intrauterine growth restriction, translocation, unbalanced

## Abstract

**Background:**

Copy number variations in chromosomal segments 3q29 and 13q34 are described in the literature, with varying reported phenotypic findings.

**Case:**

We report the case of a male proband found to have prenatal hydrocephalus and intrauterine growth restriction at 32 weeks gestation. Genetic testing revealed a gain of a 4.8 megabase (Mb) region of 3q29 and a loss of a 5.4 Mb region of 13q33.3q34, yielding partial trisomy 3q29 and monosomy 13q34. FISH studies suggest that this unbalanced translocation resulted from a paternal balanced translocation. Findings at birth included respiratory distress requiring intubation, small birth weight, hypotonia, coagulopathy, pelvocaliectasis, and macrocephaly, with MRI demonstrating severe cortical thinning and white matter volume loss.

**Conclusions:**

Though unbalanced translocations are highly individualized with varied phenotypes even among close relatives, this discussion of a proband and family members with translocation of chromosomes 3 and 13 contributes to published knowledge regarding these variants. We demonstrate the utility of online databases DECIPHER, OMIM, and Unique, as well as a translocation risk calculator, for understanding genetic anomalies and prognostic data for families affected by similar genetic variations.

**Abbreviations**
CPAP: Continuous positive pressure ventilation
DECIPHER: Database of Chromosomal Imbalance and Phenotype in Humans using Ensembl Resources
DOL: Day of life
OMIM: Online Mendelian Inheritance in Man

## 1 Introduction

Genetic translocations occur when a portion of one chromosome breaks and reattaches to a different chromosome. Translocations are a relatively common chromosomal structural abnormality, occurring in about 1 in 1000 to 2000 births (Lubs and Ruddle 1970; Warburton 1991), and most do not produce disease. In most cases, balanced translocations do not result in loss or gain in genetic information, while unbalanced translocations may yield missing or duplicated segments; both may occur de novo or can be passed from a carrier parent with a balanced or unbalanced translocation.

The terminal band of the long arm of chromosome 3, designated 3q29, contains a 1.6 megabase (Mb) segment reported to be prone to copy number variations. This region includes 21 protein‐coding genes, 3 noncoding RNAs, 3 antisense transcripts, and 1 micro‐RNA (Ballif et al. 2008; Willatt et al. 2005). Important genes in the region include p21 activated kinase 2 (*PAK2*), a signaling enzyme involved in neuron development (Kreis and Barnier 2009) and linked to social behavior (Mulle et al. 2010; Quintero‐Rivera et al. 2010; Wang et al. 2018), and Synapse‐associated protein 97 (*SAP97*), a scaffold protein found in membrane channels that regulates neuron excitability (Fourie et al. 2014). The region also contains Tyrosine kinase non‐receptor 2 (*TNK2*) signaling enzyme involved in brain development, which when overexpressed may be linked to epilepsy (Hitomi et al. 2013), and Nuclear cap‐binding protein 2 (*NCBP2*), shown to modify the effects of adjacent neurodevelopmental genes (Singh et al. 2020). Variation in reporting and lab testing classification of 3q29 abnormalities and the paucity of prognostic information for affected patients and families led to the creation of the Emory 3q29 Project, an online registry compiling data from patients with 3q29 copy number variation (Project TEq, n.d.). This database now includes more than 100 patients and has been valuable in providing support for as well as characterizing the clinical features of individuals with 3q29 variants.

Duplications of 3q29 have been reported, arising either de novo or inherited, with an estimated frequency between 1:8000 and 1:75,000 (Pollak et al. 2020). Most described patients have copy number variations that include all or a portion of the 1.6 Mb region described above (Ballif et al. 2008), though variation in breakpoints may be responsible for the wide range of phenotypes. Reported birth characteristics include early delivery (38.1 weeks compared to 39.2) and smaller birth weight by 0.74 pounds (Pollak et al. 2020) compared to control. In addition, approximately 80% of individuals with 3q29 duplication have health complications over the first year of life, including respiratory distress (29%), poor weight gain (41.9%), feeding difficulty (54.8%), and hypotonia (38.7%) (Pollak et al. 2020). In early life, social and intellectual characteristics may include cognitive disability, behavioral disruption, expressive language delay, receptive language delay, and developmental delay (Ballif et al. 2008; Pollak et al. 2020; Quintela et al. 2015; Lesca et al. 2012; Fernández‐Jaén et al. 2014), while older children have increased rates of learning disabilities and a 26‐fold increase in autism spectrum disorders compared to control (Pollak et al. 2020). Approximately 25% develop seizure disorders (Pollak et al. 2020; Fernández‐Jaén et al. 2014; Kessi et al. 2018). Physical characteristics include dental (68%) and genitourinary (19%) abnormalities, while cardiac (6%) and renal (3%) defects have been less frequently observed (Pollak et al. 2020). In one reported family of 5 individuals with inherited 3q29 duplication, all demonstrated microcephaly and intellectual disability (Lisi et al. 2008).

Abnormalities in the terminal region of the distal long arm of chromosome 13 can yield similar features. Genes in this region include SRY‐box transcription factor 1 (*SOX1*), a transcription factor with regulatory roles in development and cell fate, and Rho guanin nucleotide exchange factor 7 (*ARHGEF7*), a mediator of multiple cell functions including migration, attachment, and apoptosis (Lei et al. 2018). This region additionally includes coagulation factors 7 and 10, cell cycle mediator Cell division cycle 16 (*CDC16*), and NAD(P)HX dehydratase (*NAXD*), a metabolite repair enzyme whose deficiency has been linked with encephalopathy and febrile illness‐induced neurodegeneration (Van Bergen et al. 2019; Majethia et al. 2021). 13q deletion syndrome includes varying degrees of intellectual disability, developmental delay, growth restriction, obesity, and craniofacial dysmorphism (Reinstein et al. 2016; Huang et al. 2012), as well as congenital cardiac, brain, and renal anomalies (Huang et al. 2012). Craniofacial findings may include broad/flat nasal bridge, short philtrum, hypertelorism, and ear malformations, as well as broad necks (Huang et al. 2012; Ballarati et al. 2007; Kirchhoff et al. 2009; Quélin et al. 2009). Manifestations vary based on the specific region of chromosome 13 deleted: patients with deletion proximal to or including 13q32 tend to have severe malformations and intellectual disability, while more distal deletions in 13q33‐34 demonstrate milder intellectual disability without organ malformations (Ballarati et al. 2007; Walczak‐Sztulpa et al. 2008).

Here, we describe the clinical course and genetic evaluation of a patient with an unbalanced translocation involving the distal long arms of chromosomes 3 and 13. We discuss involved genes and review what is known about these genetic variants using online databases DECIPHER, OMIM, and Unique, and utilize a translocation risk estimation calculator to estimate prognostic data. The aim of this report is to contribute to published literature regarding chromosome 3 and 13 translocations and to provide a framework for the utilization of online resources for families affected by similar genetic variants.

## 2 Case Report

The proband was born via cesarean section at 38 weeks and 0 days to a 28‐year‐old G3A2 parent. Maternal obstetric history included two prior spontaneous abortions. The father had two siblings who had died, one due to reported trisomy 13 and another due to holoprosencephaly that could not be further characterized by the family. Prenatal care in the first and second trimesters, including ultrasound at weeks 9 and 21 and maternal non‐invasive prenatal DNA testing at week 10, had been unremarkable (UNITY Complete).

At 32 weeks, there was concern for poor fetal growth with estimated fetal weight 1 414 g; thus, the mother was referred to maternal fetal medicine for further workup and care. Ultrasound demonstrated severe bilateral ventriculomegaly and inferiorly displaced midline structures, with poor visualization of the cerebellum, 3rd, and 4th ventricles (Figure 1). Estimated fetal weight and abdominal circumference were below the 1st percentile.

**FIGURE 1:**
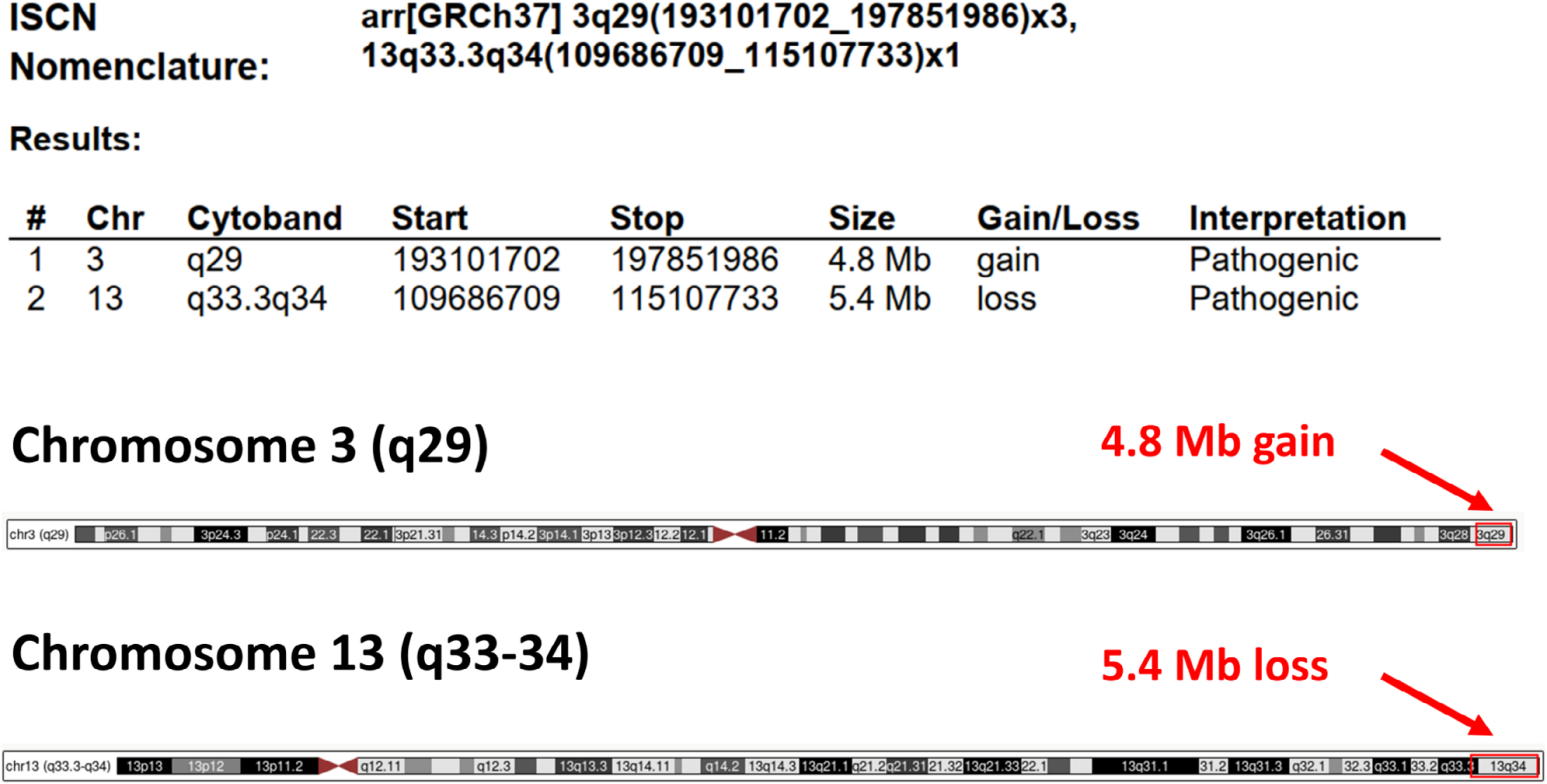
Ultrasound image of cranium at 32w2d demonstrating significant bilateral hydrocephalus. Notably, prior ultrasounds including 21w anatomy scan were unremarkable. By 35w2d the hydrocephalus had progressed, and the calvaria based estimated gestational age was consistent with a 40w1d fetus.

Fetal cells were obtained by standard methods from 30 cc amniotic fluid at 32 weeks gestation. 8 out of 22 metaphase cells were analyzed and found to have a modal number of 46 chromosomes. Giesma (GTW) banding performed on metaphase spreads according to published protocols in the AGT Cytogenetics Laboratory Manual 3rd edition (*The AGT Cytogenetics Laboratory Manual* 1997) demonstrated overall normal morphology. Karyotype analysis at the 650–850 band level did not detect any abnormalities due to the small size of these regions (Leica CytoVysion system, Leica IL, USA). Whole genome microarray using the Affymetrix CytoScan HD Microarray system, comparing the patient's sample to a control cohort of 270 individuals (Affymetrix, Santa Clara, CA, USA). Analysis was performed using Chromosome Analysis Suite software and copy number variations were determined using the human genome build 19 (hg19/NCBI build 37). Microarray demonstrated a terminal copy number gain involving chromosome 3q29 (4.8 Mb duplicated from position 19,310,702 to 197,851,986) and a terminal copy number loss involving chromosome 13 from q33.3 to q34 from 109,686,709 to 115,107,733 (5.4 Mb deleted) (Figure 2). Progressive ultrasounds were performed and by 35w2d it became evident that the hydrocephalus was non‐resolving and progressive (Figure 1). The fetus was followed weekly until delivery at 38 weeks.

**FIGURE 2:**
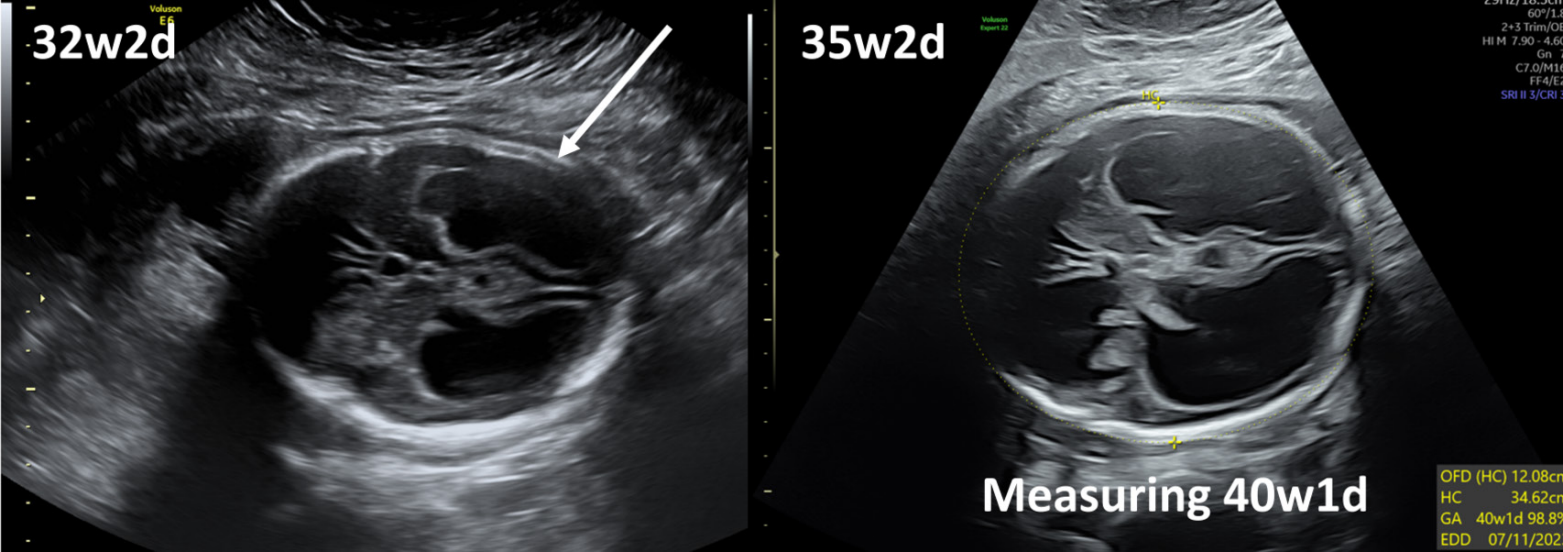
Coordinates of translocation on chromosome 3 gain and chromosome 13 loss from genetic analysis. Idiogram demonstrates the chromosomal positions of the loss and gain and highlights the acrocentric nature of chromosome 13, which may have been a predisposing factor to the translocation.

At birth by cesarian section, the neonate weighed 2712 g with Apgar scores of 4 at 1 min and 7 at 5 min. On initial examination, the neonate opened eyelids spontaneously in response to stimulation and was noted to have macrocephaly with splayed cranial sutures and an open, soft anterior fontanel. Further examination revealed an intact palate, patent nares, normal heart sounds, normal abdominal findings, and normal external male genitalia with descended testes. There were no identified rashes, petechiae, or skin lesions, and normal extremities were noted. The neonate was admitted to the NICU with respiratory distress requiring intubation and was started on inhaled nitric oxide, dopamine, hydrocortisone, ampicillin, and gentamicin. Chest xray demonstrated diffuse bilateral pulmonary opacities and a left pneumothorax, for which an emergent chest tube was performed.

Umbilical cord blood collected at the time of birth was sent for fluorescence in situ hybridization testing using subtelomeric probes for 3q and 13q, which confirmed the unbalanced translocation between chromosomes 3 and 13. Within the sample, two normal chromosome 3s, one normal 13, and a derivative 13 were identified, resulting in three copies of 3q29 to 3q terminal and a single copy of 13q34 to 13q terminal. Parental FISH studies demonstrated that these abnormalities were inherited as the result of a paternal balanced translocation.

Echocardiogram on day of life (DOL) 0 demonstrated a small interatrial septal defect and moderate patent ductus arteriosus with left‐to‐right flow. The neonate had tricuspid valve regurgitation with an estimated right ventricular pressure of 47 mmHg, with normal ejection fraction of 64%. Cranial ultrasound demonstrated severe hydrocephalus involving the lateral, third, and fourth ventricles without hemorrhage or clot, and mild left midline shift due to relative enlargement of the right lateral ventricle. Abdominal ultrasound demonstrated mild bilateral pelvocaliectasis without hepatosplenomegaly. Labs were significant for a coagulopathy with platelets 264 k, INR 2.2, PT 24.7, PTT 44, and fibrinogen 234. Total bilirubin was 4.0 on DOL 1, with sodium 126. Blood cultures were negative. The neonate received fresh frozen plasma and two doses of vitamin K with plans for serial lab monitoring. Total parenteral nutrition was provided until DOL 2, when breast milk feeds were added through a nasogastric tube.

On DOL 3, the left chest tube was removed, and non‐contrasted brain MRI demonstrated severe thinning of the right cortical mantle, severe bilateral cerebral and cerebellar white matter volume loss, hemosiderin staining lining both ventricles and the germinal matrix, and a small volume hemorrhage within the left ventricle. After extensive discussion with the patient's family regarding the unknown prognosis of chromosome 13 to 3 translocation, he was extubated to CPAP on DOL 7 and weaned to HFNC over the following days. Cranial findings on imaging remained stable. The patient was discharged home on DOL 18 with hospice support and home oxygen. On the day of discharge, the physical exam was unchanged, and the neonate moved all extremities in response to stimuli with global reduction in tone.

## 3 Conclusions

We describe the genetic features and pre‐ and post‐natal clinical course of a patient with a 4.8 Mb duplication involving 3q29 and a 5.4 Mb deletion of 13q33.3 to 34, suggestive of an unbalanced translocation. FISH analysis revealed that a paternal balanced translocation was likely the source of his genetic aberration. In obtaining parental history, the father of the baby reported two deceased siblings, one of whom had trisomy 13 and one with an unspecified holoprosencephaly. Though genetic testing on the paternal grandparents was not available, it is likely that this neonate's father inherited a balanced translocation of chromosome 13 while the paternal uncle inherited the unbalanced complement with duplication in 13 and deletion in 3. The paternal sibling with holoprosencephaly may also have had chromosome 13 duplication, as holoprosencephaly is strongly associated with chromosome 13 copy number variations (Folkerth and Lidov 2012; Bellucco et al. 2019). We also note that the two prior miscarriages reported may have been related to inherited translocations. Chromosomal translocations lead to widely varied phenotypes even among close relatives depending on the specific combination of genes inherited, as demonstrated in this family.

The clinical findings observed in our case may be multifactorial; while some features result directly from gain or loss of genetic information, it is likely that indirect dosage‐sensitive interactions and altered regulatory functions also contribute to phenotypic findings. Some clinical features of this neonate are consistent with those described for 3q29 duplication syndrome including small birth weight, respiratory distress, feeding difficulty, and hypotonia (Pollak et al. 2020), though this neonate does not demonstrate other reported 3q29 duplication features, notably microcephaly and high palate (Pollak et al. 2020; Quintela et al. 2015; Lesca et al. 2012). Neurological features are among the most commonly reported with both 3q29 duplication (Pollak et al. 2020; Fernández‐Jaén et al. 2014; Kessi et al. 2018) and 13q33‐34 deletion (Reinstein et al. 2016; Huang et al. 2012). While many cases of 3q29 copy number variation have microcephaly (Cuschieri et al. 1977; Kučerová et al. 1975), this patient exhibited macrocephaly and hydrocephalus, possibly as a result of intracranial hemorrhage with obstruction as evidenced by hemosiderin deposition along the germinal matrix noted by MRI.

Several free web‐based resources contain valuable information for families and researchers wanting to learn more about specific genetic variations, organizing what is known about each gene from human, animal, and molecular studies. We investigated specific genes involved in this case first using the online DECIPHER (Database of Chromosomal Imbalance and Phenotype in Humans using Ensembl Resources; DECIPHER v11.24: Mapping the clinical genome (deciphergenomics.org)) project (Harrison et al. 2024; Firth et al. 2009) and OMIM (Online Mendelian Inheritance in Man) databases (https://www.omim.org/ 2024). This patient's chromosome 3 duplication involves 4.8 Mb with 78 known genes, 40 of which are listed in OMIM. The 4.8 Mb region fully encompasses and extends beyond the 1.6 Mb segment reported to produce 3q29 duplication and deletion syndromes (Pollak et al. 2020; Sanchez Russo et al. 2021). Of 23 protein‐coding genes listed in OMIM with 3q29 duplication, all of which are duplicated in our patient, 8 are associated with a known disease phenotype (“morbid” genes on OMIM): *CEP19*, *DYNLT2B*, *NRROS*, *PAK2*, *PCYT1A*, *RNF168*, *SLC51A*, and *TFRC*. Genes associated with central nervous system abnormalities included Negative regulator of reactive oxygen species (*NRROS*), variation of which has been shown in 9 children who exhibited hypotonia, spasticity, and seizures, with marked cerebral atrophy and ventricular enlargement early in life (Smith et al. 2020; Dong et al. 2020). In mice, *NRROS* deficiency led to progressive neurodegeneration and early death, with evidence that *NRROS* is important in microglial homeostasis during brain development (Wong et al. 2017). Ring finger protein 168 (*RNF168*) variants have been linked to DNA double‐strand breaks, cell cycle checkpoint abnormalities, immunodeficiency, and microcephaly (Devgan et al. 2011; Stewart et al. 2007). PAK2 on 3q29 has been previously suggested to be a candidate responsible for intellectual delay and disability in individuals with 3q29 copy number variation (Streata et al. 2020). *DLG1*, encoding a scaffolding protein with roles in neuron signaling (Walch 2013) and synapse function (Fourie et al. 2014), and *BDH1*, encoding a catalyst of fatty acid metabolism (Newman and Verdin 2017), are suggested to be critical genes for 3q29 duplication syndrome (Bauleo et al. 2023; Tassano et al. 2018). Of the 18 OMIM‐indexed genes in the duplication outside of the 3q29 region, 3 have associated disease phenotypes: *ATP13A3* with pulmonary arterial hypertension (Gräf et al. 2018), *RPL35A* with Diamond‐Blackfan anemia (Farrar et al. 2008), and *RUBCN* with spinocerebellar ataxia (Assoum et al. 2013; Seidahmed et al. 2020).

The deletion in 13q33.3q34 contains 79 genes, 28 of which have OMIM entries with 13 “morbid” genes linked to disease phenotypes (*LIG4, CHAMP1, NAXD, CARS2, IRS2, COL4A1, COL4A2, ING1, ATP11A, F7, F10, PROZ, GRK1*). *CHAMP1* variation is associated with intellectual disability, language delay, hypotonia, and behavioral abnormalities (*Nature* 2015; Hempel et al. 2015; Garrity et al. 2021; Isidor et al. 2016; Levy et al. 2022). NAD(P)HX dehydratase (*NAXD*) variants have been demonstrated in several patients with progressive early‐onset encephalopathy and brain edema (Van Bergen et al. 2019; Borna et al. 2020); loss of enzyme function leads to destructive accumulation of toxic NADH metabolites in the brain and heart (Van Bergen et al. 2019). Cysteinyl‐tRNA synthetase 2 (*CARS2*) variation is reported in a series of patients with abnormal mitochondrial oxidative phosphorylation resulting in variable severity of seizures, hypotonia, psychomotor delay, and progressive cortical and cerebellar atrophy (Coughlin et al. 2015; Hallmann et al. 2014; Samanta et al. 2018). Several genes in this region have roles in neuronal development and have been identified as potential “critical genes” for microcephaly (Kirchhoff et al. 2009), including transcription factor *Sox1 (*Kan et al. 2004; Pevny et al. 1998), signaling protein *ARHGEF7 (*López Tobón et al. 2018; Häbig et al. 2013), and mRNA regulator *UPF3B (*Alrahbeni et al. 2015).

Genetic counseling is an important aspect of this case, as several family members and pregnancies were likely affected. Though translocations are highly variable, prognostic data can be inferred using an online risk estimation calculator published by Trunca, Mendell, and Schilit in 2022 (Trunca et al. 2022). This resource is based on logistic regression models created from the genetic data of more than 6000 individuals with balanced translocations from more than 1000 families (Trunca et al. 2022). Using input data from this case with chromosomes 3q29 and 13q33, male carrier, ascertained by a child with unbalanced chromosomes, the calculator estimates a 28.46% risk of miscarriage and an 18.16%–32.35% risk of a child with unbalanced chromosomes. Given that this was a paternally inherited variation with multiple family members affected, the parents were counseled regarding their risk of future miscarriages or offspring with birth defects including hydrocephalus or IUGR, and a genetics referral was made. The Unique database (rarechromo.org) is an additional resource for investigating whether specific genetic variants have been reported, and for families to connect with others with similar variations. Cases reported in the database include 46,XX.ish der(13)t(3;13)(q29;q34) (Code number 11763), 46,XX,der(13)t(3;13)(q29;q34)de novo (Code number 16516), arr 3q26.31q29x3 13q33.3q34x1 (Code number 64929), and arr 3q26.32q29x3 arr 13q34x1 (Code number 69250) (https://rarechromo.org 2024).

This report describes the clinical course of a patient with a novel 4.8 Mb gain including 3q29 and 5.4 Mb loss of 13q33.3q34, with FISH studies indicating paternal inheritance from a balanced translocation. The patient's clinical findings included intrauterine growth restriction, hydrocephalus, macrocephaly, hypotonia, small birth weight, and respiratory distress requiring intubation. This patient was discharged with home oxygen and hospice support on DOL 18. This patient's two regions of copy number variation include 157 known genes, 68 of which have OMIM entries, with 20 known to have associated disease phenotypes. Using online databases including DECIPHER, OMIM, Unique, and a translocation risk estimation calculator, we reviewed the indexed genes and reported variants of these regions, discussing several which may have contributed to this proband's features. This case and discussion contribute to published literature regarding chromosome 3 and 13 copy number variation and provide insight into a previously undescribed translocation, which may be useful prognostic data for families with similar genetic variations.

## Author Contributions

All authors reviewed and agreed to the final manuscript. Teresa E. Fowler: Conceptualization, writing original draft, editing. Ryan F. Bloomquist: Conceptualization, review, editing. Anthony R. Gregg: Conceptualization, review, editing.

## Ethics Statement

The authors have no conflict of interest to declare. No funding was received for this work. All authors contributed significantly to this work, read and approved the final versions of the manuscript. Consent for this work was provided by the patient guardians, and the work has been de‐identified.

## Consent

Written consent to publish this de‐identified patient information was provided by the parents.

## Conflicts of Interest

The authors declare no conflicts of interest.

## Data Availability

Data sharing is not applicable to this article as no new data were created or analyzed in this study.
